# Anxiety and Depression in a Non-Clinical Sample of Young Polish Adults: Presence of Meaning in Life as a Mediator

**DOI:** 10.3390/ijerph19106065

**Published:** 2022-05-17

**Authors:** Małgorzata Szcześniak, Adam Falewicz, Klaudia Strochalska, Radosław Rybarski

**Affiliations:** 1 Institute of Psychology, University of Szczecin, 71-017 Szczecin, Poland; adam.falewicz@usz.edu.pl (A.F.); klaudiastrochalska@tlen.pl (K.S.); 2 Institute of Psychology, John Paul II Catholic University of Lublin, 20-950 Lublin, Poland; radekrybarski@kul.lublin.pl

**Keywords:** anxiety, depression, meaning in life, presence of meaning, searching for meaning, mediation, early adulthood

## Abstract

Early adulthood, between 18 and 25, is viewed as a decisive period of life for the prevention and treatment of anxiety and depression. Although the topic of their mutual relationship is well-known, little has been uncovered about the mechanism underlying this connection. To understand the indirect pathways between anxiety and depression, we chose the sense of meaning of life as a mediator because people’s beliefs that their lives are or can be purposeful may protect against depression. The sample was composed of 277 Polish young adults. A small majority of the participants were women (58.8%). The mean age was *M* = 22.11 (*SD* = 1.72). We used in the research the Direct Behavior Rating-Scale Items Scale, the Meaning in Life Questionnaire, and the Brief Screen for Depression. Correlational analysis showed that, consistent with past findings, anxiety correlated positively with depression and searching for meaning. It was also negatively associated with presence of meaning. Moreover, depression was negatively linked to presence of meaning and positively with searching for meaning. Regression-based mediation analyses (PROCESS macro 3.4) proved that the relationship between anxiety and depression was mediated by presence of meaning in life, suggesting that having a sense of meaning may be a pathway by which feelings of tension relative to adverse events protect against depression.

## 1. Introduction

Early adulthood, between ages 18 and 25 [1], is considered a crucial stage of life for the prevention and treatment of anxiety and depression [2,3]. During this period, young adults intensively consolidate their identity [4,5] and experience complex challenges associated with leaving their parents’ house, independent living, and starting higher education, a full-time job, or their own family [6,7,8]. These developmental tasks and the uncertainty related to new experiences of the transition to adulthood may contribute to a sense of anxiety leading, in turn, to depression. In fact, according to research conducted by Goodwin et al. [9], over the past 11 years (2008 to 2018), American respondents aged 18 to 25 had the highest, almost twofold, increase in the level of anxiety compared to other age groups. Moreover, the outcomes of the Great Smoky Mountains Study [8] confirmed that anxiety is much more prevalent than had formerly been alluded to and that the most evident increment in anxiety is associated with the passage to emerging adulthood. Likewise, Twenge et al. [10], based on a nationwide representative survey of U.S. young adults of the same age, found that the occurrence of a major depressive episode increased by 7 percentage points between 2009 and 2017.

Emerging adulthood is also a time when meaning in life is considered an important developmental task [11] that plays a crucial role in the context of mental health [12]. Comprehensive analyses suggest that young adults experience various life-changing events that require from them the presence of meaning and looking for meaning [13], especially when they feel anxiety or despondency.

Despite the importance of young adulthood for human development, a lack of studies that explicitly address the topic of health and well-being during this stage of life can be observed [1]. Therefore, the rationale for undertaking the current analysis was the paucity of research [14] on the direct relationship between anxiety and symptoms of depression in a non-clinical sample of young adults. Moreover, the experience of anxiety and depression in early adulthood may hinder the developmental achievements associated with graduating from high school or university, establishing deeper interpersonal relationships, and entering the labor market [15].

Additionally, we were interested in whether the association between the abovementioned constructs was mediated by other psychological variables. In fact, Jacobson and Newman underlined that “little has been uncovered about the mechanism underlying this connection” [16] (p. 66). Moreover, other researchers [17,18] have emphasized the need to understand not only the direct relationship between anxiety and depression but also its indirect pathways. To achieve this goal, we chose the sense of meaning of life since people’s beliefs that their lives are or can be purposeful [19] may protect against depression.

### 1.1. Anxiety and Depression

There is some evidence that the constructs of anxiety and depression overlap within clinical and normal samples [20], as they both share negative affectivity [21,22] and are related to stressful life conditions [23]. Nevertheless, there is also a substantial body of research that confirms a deep-rooted difference between anxiety and depressive symptoms on distinct levels [21,24,25]. Nowadays, a more subtle perspective represented by the dual-construct theory considers anxiety and depression as separate but coexisting constructs [21,26,27].

Anxiety is recognized “as an anticipatory state of active preparation for dealing with threat” [28] (p. 837). Several studies based on cognitive theories have provided strong evidence that anxiety is the result of increased processing of danger-related information [29,30,31] and enhanced tension [28,32]. It prevalently refers to prospective and possibly negative events [25] and circumstances that have the likelihood to cause specific future problems [23]. In turn, depression is a long-term mood disorder [33] related to adverse life events [34] and different kinds of loss episodes [20], such as the loss of a loved one [20,34,35], a close relationship [36,37], physical health [20,38], or a job and financial stability [39,40,41,42]. Eysenck et al. [20] drew special attention to two aspects of loss. They allude to the valuable nature of someone or something and imply the impossibility of retrieving the lost reality.

According to different studies, both anxiety [43,44] and depression [45,46,47,48] arising in the early stages of development tend to peak and persist in early adulthood. The relationship between anxiety and depression in young adults has been analyzed from several perspectives [45]. Regardless of the adopted approach, both disorders tend to be closely related [25,49,50]. For example, in a recent study of Chinese medical students, Shao et al. [51] found a positive correlation between anxiety and depression (*r* = 0.403 **). Similarly, other researchers have reported that anxiously attached [52,53] and socially anxious [54] people report higher levels of depression. Moreover, a growing body of epidemiological and clinical evidence shows that anxiety often predates depression [18,55,56]. Anxiety was also a significant predicting factor of depression in people with multiple sclerosis [57]. Anxiety predicted depression in a longitudinal study among American adolescents [27]. Taking into account the results of the research conducted so far on the relationship between anxiety and depression, we adopted the following hypothesis:

**Hypothesis** **1** **(H1).** *Anxiety positively correlates with depression.*

### 1.2. Anxiety and Meaning in Life

Consistent with the cognitive approach, anxiety disorders arise from a real or distorted viewpoint of danger [58,59] and biased interpretation of ambiguity [60]. Subjective perceptions of threat or risk usually make people want to understand what is happening in their lives and what the consequences of these events may be [61]. According to the conservation of resources theory [62], when confronted with a difficult reality, individuals try to acquire, preserve, defend, and foster different resources to cope with unexpected and aversive situations. Therefore, people who tend to be anxious may look for meaning in life because struggling to understand one’s life may bring with it a re-evaluation of stressful life conditions [61], adaptation to the required changes [63], and protection of mental health [64].

Although meaning in life can be of great significance at any stage of development, Dezutter et al. [65] suggest that it is especially important in early adulthood when young people face new challenges and are anxious to meet life requirements. There are different perspectives on meaning in life [66,67]. The one adopted for the current study alludes to Steger’s approach, which draws a distinction between having (presence) and seeking (searching) meaning in life [66]. The dimension of presence refers to people’s comprehension of themselves and the world around them. The dimension of searching relates to efforts made to achieve such understanding.

Recent research [19] has shown that respondents reporting higher levels of anxiety also declared higher searching for meaning and lower presence of meaning. Moreover, Yek et al. [68] observed a negative correlation between health anxiety and presence of meaning and an inverse association with search for meaning in life. Slightly different results have been obtained by researchers who investigated the relationship between specific types of anxiety and overall meaning in life. For example, Zhang et al. [69] found that older Chinese adults feeling threatened by death expressed lower presence of and searching for meaning. Likewise, Ardelt [70] confirmed that White and African American elderly respondents who acknowledged a higher degree of fearful and avoidant attitudes toward death denoted lower levels of purpose in life as well. Although the results seem inconclusive, due to the different meanings given to the dimension of searching for life, we posited that:

**Hypothesis** **2** **(H2).** *Anxiety negatively correlates with presence of meaning in life and positively with searching for meaning.*

### 1.3. Meaning in Life and Depression

The review of studies has shown quite unequivocally that higher levels of meaning are negatively associated with depression [71]. Steger et al. [19] observed in a sample of young people between 18 and 24 that depression correlated negatively with presence of meaning (*r* = −0.53 ***) and positively with search for meaning (*r* = 0.25 ***). In another study [71], participants’ presence of meaning was significantly and negatively related to depressive symptoms for both women and men. Instead, search for meaning was significantly correlated with depression only in the case of women. Moreover, Yu et al. [71] found that presence of meaning was a predictor of depression in men. Likewise, Korkmaz and Güloğlu [61], based on a study conducted in Turkey during the COVID-19 pandemic, confirmed that meaning in life significantly predicted depression among adult respondents. Mascaro and Rosen [72] found evidence that undergraduate students with greater meaning tend to report fewer depressive symptoms than their counterparts with a lower level of meaning. In turn, lack of meaning was found to relate to psychopathology [73]. For example, patients with depression felt their lives to be meaningless and worthless, expressing destructive thoughts or attempting suicide [74].

The presented findings coincide with the consistent pattern encountered in previous empirical and clinical research where meaning in life plays a predictive role in mental health and overall well-being [65,74,75,76]. This role results from the intrinsic nature attributed by various authors to the sense of meaning in life. People strive to understand who they are and the purpose of their lives [65,77]. They derive a sense of life from various sources along their life span [78]. An absence of meaning does not fulfill people’s need for it and may lead to frustration. For example, Dezutter et al. [65] suggested that a lack of meaning may drive early adults to internalize depressive symptoms and unhealthy outcomes. Using cluster analysis, the authors found that emerging adults in the low presence–low search profile were the most poorly adapted, showing maladaptive psychosocial functioning. When people judge their lives as devoid of content, they either start looking for meaning or plunge further into frustration [79]. In the latter case, such persons may experience the phenomenon of existential vacuum. In this context, Frankl [77] spoke of depression, stressing that it cannot be understood without a feeling of meaninglessness. In line with earlier studies, we hypothesized that:

**Hypothesis** **3** **(H3).** *Depression negatively correlates with presence of meaning in life and positively with searching for meaning.*

### 1.4. Meaning in Life as a Mediator

Several studies have identified different pathways by which anxiety may elicit depression: avoidance [27]; loneliness [17]; perceptions of close, peer, and group relationships [16,80]; anhedonia [18]; repetitive negative thinking [81]; and professional quality of life [82].

Because we have not found any other studies in which the meaning in life would mediate the association between anxiety and depression, the results obtained by Marco et al. [83] can shed some light on this relationship. In their study, authors focused on the mediation of purpose in anxiety before and after treatment and depression before and after treatment. Through the longitudinal measurement, the researchers found that meaning in life tends to reduce both anxiety and depression symptoms during cognitive behavioral therapy (CBT). Patients who were practicing meaning making showed lower levels of anxiety and depression after CBT. This allows us to suppose that meaning in life may act as a mediator that explains the change in the level of psychopathology before and after the therapy. We believe that, combined with the extensive literature on the temporal sequelae of anxiety and depressive disorders, this is a sufficient line of reasoning to justify the mediating role of meaning in life in cross-sectional research between both adjustment disorders.

Moreover, according to terror-management theory, people can overcome their anxiety when they feel that their lives are permeated with meaning [84]. In turn, low meaning in life is considered a vulnerability factor for depression, as people with a high level of anxiety may find it more difficult to discover authentic meaning and, consequently, may be more prone to developing depressive symptoms. For example, among patients diagnosed with depression, it has been noted that higher levels of meaning making were related to subsequent reductions in depression [85]. Moreover, searching for meaning is helpful in unfavorable situations [86]. Additionally, researchers observed that meaning making was related to less discrepancy between beliefs and goals, which was related to fewer instances of depression [87]. Based on the above research, we expected that:

**Hypothesis** **4** **(H4).** *Presence of meaning and searching for meaning act as mediators between young adults’ anxiety and their symptoms of depression.*

## 2. Materials and Methods

### 2.1. Participants and Procedure

The sample was composed of 277 Polish young adults. A small majority of the participants were women (58.8%). The mean age was *M* = 22.11 (*SD* = 1.72). Respondents were recruited primarily through social networking websites (e.g., Facebook) in October 2021. A convenience sample was used due to the greater availability of participants and the simplicity of the process. The main inclusion criterion for participation in the study was age between 18 and 25, which is characteristic of early adulthood. The exclusion criterion was the lack of diagnosis of a clinical form of depression. The participants were contacted by the study team. After completion of the study, access to the connection between the link and the interactive battery was closed. The obtained raw data have been stored since then in the form of matrices on a private storage medium. The participants were expected to complete questionnaires in one session and were informed about the purpose of the research, its confidential character, and the possibility of withdrawing from the study at any time during its duration. Informed written consent was obtained after the participants were familiarized with the goal of the study.

### 2.2. Direct Behavior Rating-Scale Items Scale

The Direct Behavior Rating-Scale Items Scale (DBR-SIS), developed by von der Embse et al. [88] and translated into Polish by Zarzycka et al. [89] is a tool used to estimate the participants’ level of anxiety. The scale includes three items that allude to different facets of anxiety: social (“I am worried what others will think”), cognitive (“I feel restless”), and physiological (“I am nervous”). The items range from 1 to 10, where 1 = no anxiety, and 10 = very high anxiety. In previous studies, the Cronbach’s alpha value was 0.79 [89]. In the present study, the internal consistency coefficient was very good, with α = 0.87.

### 2.3. Meaning in Life Questionnaire

The Meaning in Life Questionnaire (MLQ), created by Steger and colleagues [90] and adapted into Polish by Kossakowska et al. [91], consists of 10 statements that reflect two dimensions of meaning in life. Presence of meaning pertains to the belief that one’s existence is valuable and significant (e.g., “I have discovered a satisfying life purpose”). Searching for meaning refers to a commitment to looking for sense (e.g., “I am seeking a purpose or mission for my life”). Participants answer on a Likert 7-point scale (1 = absolutely untrue, and 7 = absolutely true). The MLQ is considered a reliable measurement of meaning in life, showing very good Cronbach’s alpha values between 0.86 and 0.90 [91,92]. In the current study, the subscale “presence of meaning” had α = 0.88, and the subscale “searching for meaning” had α = 0.87.

### 2.4. Brief Screen for Depression

Brief Screen for Depression (BSD), developed by Hakstian and McLean [93] and translated into Polish by Zarzycka et al. [89], is a concise questionnaire designed to assess non-psychiatric patients for signs of depression. The BSD contains four items (e.g., “How many times during the last two days have you been preoccupied by thoughts of hopelessness, helplessness, pessimism, intense worry, unhappiness, etc.?” [93] (p. 140). A cut-off score of 21 may denote a clinical level of depression. According to Lipps and Love [94], the BSD shows acceptable degrees of internal consistency between α = 0.63 and 0.65. In the current study, Cronbach’s alpha was equal to 0.75.

### 2.5. Statistical Analysis

The analysis of the statistical data was performed with IBM SPSS Statistics version 20 (Armonk, NY, USA). The variables of anxiety, presence of meaning, searching for meaning, and depression were tested for the normality of distribution. Indices of skewness and kurtosis lower than ±2 were assumed to verify a normal univariate distribution [95]. The Variance Inflation Factor (VIF) was applied to diagnose the degree of collinearity between predictors in a regression model, with a coefficient higher than 5.0 as a sign of multicollinearity. A tolerance value lower than 0.1 to 0.2 was assumed to show a serious collinearity issue [96,97]. The Mahalanobis and Cook’s distance were used to detect potentially misleading outliers [98]. The Mahalanobis distance was calculated using the chi-square (χ^2^) with 5 degrees of freedom and *p* < 0.001. A Cook’s distance value larger than 1 was considered troublesome [99].

Stepwise regression was applied to scrutinize the data for a potential confounding problem. Two sociodemographic variables (sex and age) were selected in the first block. Three predictors (anxiety, presence of meaning, searching for meaning) were enclosed in the second block. Substantial empirical evidence shows that anxiety [3,100] and depressive symptoms [101] are more pronounced in women than in men. According to LaFreniere [102], girls already rate themselves as more apprehensive, shy, and cautious than boys from childhood. When it comes to meaning in life, there is some support that females reveal higher intensity of purpose [103] than males. Moreover, Steger et al. [19] specified that during emerging adulthood, women tend to report higher levels of searching for meaning than men. With respect to age differences in anxiety [104,105] and meaning in life [19], the literature does not report consistent conclusions. For example, Essau et al. [105] observed that anxiety is more persistent in older adolescents than younger ones. Dezutter et al. [65] reported that although meaning in life is salient during the whole life span, it seems to be particularly important among adolescents and young adults, where they search for, discover, develop, and strengthen their identity.

The two mediation models (anxiety → presence of meaning in life → depression; anxiety → searching for meaning → depression) and the significance of indirect effects were assessed with 95% percentile confidence intervals (CI), using the 5000 bootstrapped samples procedure (Hayes PROCESS macro 3.4., Model 4) [106].

## 3. Results

### 3.1. Descriptive Statistics

The descriptive statistics (mean, standard deviation, skewness, and kurtosis) for anxiety, presence of meaning in life, searching for meaning, and depression are summarized in Table 1.

The skewness and kurtosis values ranged from −0.188 to 0.967, indicating a relatively normal distribution within the acceptable ±2. Accordingly, a Pearson correlation analysis was run.

### 3.2. Correlations

The results of the Pearson analysis (Table 2) showed statistically significant (*p* < 0.001) correlations between anxiety, presence of meaning, searching for meaning, and depression. Consistent with past findings, anxiety correlated positively with depression (H1) and searching for meaning (H2). It was also negatively associated with presence of meaning (H2). Moreover, depression was negatively linked to presence of meaning and positively with searching for meaning (H3). Interestingly, presence of meaning correlated positively with searching for meaning. The Pearson correlation coefficients found were between small and large.

Based on the results of the present study, it can be concluded that as the unpleasant feelings of tension increase in young adults, so do the levels of searching for meaning and depressive symptoms. Moreover, higher levels of anxiety are associated with lower levels of presence of meaning. Simultaneously, the higher the magnitude of depression, the greater the efforts to find understanding of meaning and the lower the intensity of presence of meaning.

### 3.3. Multicollinearity and Confounding Variables

The findings of the multiple regression analysis showed a VIF of 1.03–1.36, thus specifying that the explanatory variables were moderately associated one with another. The values for tolerance ranged from 0.737 to 0.996, being higher than the recommended 0.2. Both results imply that there was no near-linear dependence among the predictor variables. From the screen for multivariate outliers through the calculation of the Mahalanobis distance, it emerged that there were no outliers in the data. In fact, the lowest value of *p* was equal to 0.01107 (*p* > 0.001). Furthermore, Cook’s distance had scores between 0.000 and 0.066, confirming that all observations were below the suggested cut-off of 1. The analysis accounting for confounding effects confirmed that sex and age were not relevant confounders as they explained only 1.5% of the variance (*R*^2^ = 0.015) (sex: *β* = −0.011, *t* = −0.242, *p* = 0.809, and age: *β* = 0.004, *t* = 0.081, *p* = 0.936). Anxiety and both dimensions of meaning in life from Step 2 explained 40.5% of the remaining variance even after statistically controlling for the effect of sex and age.

### 3.4. Mediation Analyses

**Figure 1:**
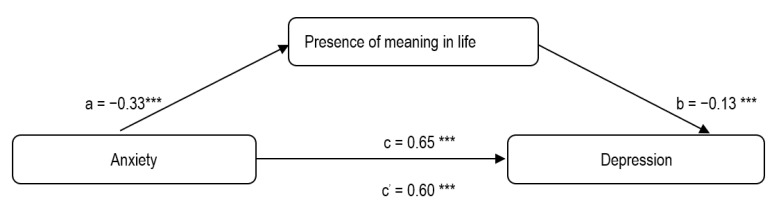
Results of mediation analysis of presence of meaning in life in the relationship between anxiety and depression. *** *p* < 0.001.

As regards H4, only the first model (anxiety → presence of meaning → depression) showed a significant effect (*p* < 0.001), with path a (*β* = −0.33) between anxiety and presence of meaning and path b (*β* = −0.13) between presence of meaning and depression (Figure 1). With the insertion of presence of meaning as a mediator, path c (*β* = 0.65) dropped to path c’ (*β* = 0.60), having the same significance value of *p*. The total indirect effect of anxiety on depression was B (SE) = 0.0425 (0.0206) with 95% CI (0.0065; 0.0884), demonstrating that the relationship between anxiety and depression was mediated by presence of meaning.

**Figure 2:**
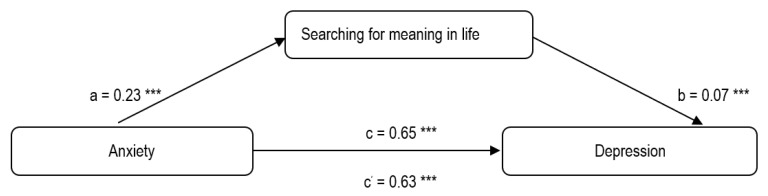
Results of mediation analysis of searching for meaning in life in the relationship between anxiety and depression. *** *p* < 0.001.

With respect to the second model (anxiety → searching for meaning → depression), the results showed an insignificant effect, with a significant path a (*β* = 0.23) between anxiety and searching for meaning and an insignificant path b (*β* = 0.07) between searching for meaning and depression (Figure 2). Moreover, the addition of searching for meaning as the mediator presented only a slight decrease of the value *β* = 0.65 (path c) to the value *β* = 0.63 (path c’). The total indirect effect of anxiety on depression was B (SE) = 0.0171 (0.0120) with 95% CI (−0.0028; 0.0439), showing the lack of mediation effect of searching for meaning.

## 4. Discussion

The present study had a fourfold purpose to examine: (1) the relationship between anxiety and depression; (2) the association between anxiety and presence of/searching for meaning; (3) the character of the correlation between presence of/searching for meaning and depression; and (4) the mediatory effect of presence of/searching for meaning between anxiety and depression. To the best of our knowledge, the current study is the first in the Polish psychological context to directly assess the topic of meaning in life as a potential mediator between anxiety and depression. The outcomes provide clear evidence about the correlational relationships of the investigated variables. Regarding the mediational models, only presence of meaning acted as a mediator between anxiety and depression. The model including search of meaning as a mediator did not find sufficient justification.

In regard to hypothesis (H1), the positive correlation between anxiety and depression is consistent with the previous literature [107,108,109], from which it emerges that greater levels of anxiety are associated with higher levels of depressive symptoms. Although the strength of the relationship in the present research (*r* = 0.62 ***) is slightly different than in other studies (e.g., *r* = 0.68 ***, [110]; *r* = 0.64 *** [111]), it still presents a large effect, lending support that both clinical-related constructs share some characteristics for mutual association.

With respect to hypotheses (H2) and (H3), the negative association between anxiety (H2)/depression (H3) and presence of meaning and the positive association between anxiety (H2)/depression (H3) and searching for meaning are in line with some anterior research findings. For example, people who declare being happier [112] show higher levels of general well-being [113] and life satisfaction [114,115,116], have a greater sense of control [117], feel more engaged at work [113,118], and believe that their lives are meaningful. Moreover, Pinquart [119] suggested, based on a meta-analysis of 70 studies, that experiencing a purpose in life, even differently operationalized, tends to be negatively correlated with depression. Considering that the dimensions of well-being positively correlate with presence of meaning, it is understandable that anxiety and depression, as opposed to the state of comfort and relief, are negatively associated with making sense of life. Furthermore, Steger and colleagues pointed out that search for meaning positively correlates with feelings of distress [120,121] and dysfunction [90] and is negatively associated with well-being, defined as both life satisfaction and feelings of happiness [120]. Therefore, the above-presented outcomes clarify the positive correlation obtained in the current study between searching for meaning and anxiety/depression. In fact, it has been found that different forms of anxiety may stimulate people to seek meaning in life [3]. This is in line with the deficit-correcting hypothesis [121], which maintains that searching for meaning mostly originates from a shortage or lack of meaning. Once the meaning is reestablished, the search would decline.

As concerns hypothesis (H4), only presence of meaning plays a mediating role in the direct relationship between anxiety and depression. It seems that making sense of life is not indifferent for people struggling with anxiety because when they discover such meaning, they may consider their experience as significant despite the difficulties. This, in turn, may lower their levels of depression. Indeed, people that experience more meaning in their lives tend to develop less severe depression symptoms [122,123,124]. With presence of meaning in life, the experienced anxiety gains a cognitive framework and prevents the development of dysfunctional cognitive schemas that could eventually lead to the unfolding of depression [125,126].

## 5. Limitations

There are several limitations to this study. To start with, its cross-sectional and correlational character prevents us from inferring any causal relationships between the measured variables. Furthermore, our research group was limited to emerging adults. Knowing that the occurrence of anxiety and depression is not restricted to this age group, it would be advisable to extend the research to participants representing the entire life span in future studies. Next, the study was conducted among Polish respondents. On the one hand, the results enrich the existing knowledge with a new cultural context and, on the other hand, prevent them from being generalized to the global population. Moreover, we included only sex and age as confounding variables, which precludes strong conclusions. We acknowledge that proposing other possible confounding variables (e.g., the presence of comorbidities, family situation such as parents’ divorce or history of alcohol abuse, the level of satisfaction with studies or work, private and professional failure) could increase the credibility of the research. However, we assumed that asking for such aggravating factors could constitute another variable that generates temporary anxiety or depressed mood. The way of responding to the questionnaires could result more from the situationally experienced anxiety or depression than from the generalized level of these variables.

Since the study was conducted during a pandemic, the underlying conditions of insecurity may have influenced the way people conceptualize meaning in life. Culture also plays a significant role. In societies where mental health is stigmatized [127], people tend to internalize and somatize mental distress. Therefore, in future studies, it would be advisable to consider also other confounding variables that are related to the pandemic, social support, negative life events experienced, cultural aspects, current geopolitical tension, schooling or studying, socioeconomic level, occupation, disability, and family relationships or support. All of these factors may affect the perception of life satisfaction and psychological resilience.

## 6. Conclusions

The present study suggests that presence of meaning in life may be a pathway by which feelings of tension relative to adverse events induce depression. In other words, a belief that one’s own life matters may act as a buffer against threatening thoughts or situations. Promoting presence of meaning and its protective role against depression might be a valuable input for therapeutic purposes. The search for greater meaning is also advised for those who have obtained a satisfactory current level of meaning.

In addition to the theoretical conclusions and practical implications resulting from our research, we propose several topics that could be used in future studies. Given that, in the current research, we used brief questionnaires designed to assess non-clinical patients for signs of anxiety and depression, it is advised to extend the study to clinical samples with patients diagnosed with serious anxiety and depression and the use of some other measurements. Such a procedure might help to verify the universal character of the mediating role of presence of meaning in clinical groups. Moreover, the evidence provided in our cross-sectional study about the mediating role of presence of meaning may serve as a rationale for an experimental design in which meaning-promoting training for people experiencing elevated levels of anxiety and depressive symptoms could be a factor that prevents greater clinical manifestations from unfolding.

Likewise, the lack of a mediating role of search for meaning in the relationship between anxiety and depression may suggest that further research is needed to control for the initial level of presence of meaning. This is justified by some previous research [128] where search for meaning was associated with higher well-being rather than distress. The outcomes of searching for meaning might also differ depending on the person’s reasons for searching, actual level of meaning in life, and personality traits [66].

Finally, due to the different emotional consequences of the search for meaning in both Eastern and Western countries, intercultural studies are suggested to verify whether the mediation effect is not displayed in a more individualistic context (than the Polish one), where meaning seeking would be more strongly associated with lower well-being [129].

## Figures and Tables

**Table 1:** Descriptive statistics for anxiety, presence of meaning, searching for meaning, and depression (*N* = 277).

Variables	*M*	*SD*	Skewness	Kurtosis
Anxiety	9.15	6.88	0.967	−0.208
Presence of meaning	25.69	7.63	−0.785	−0.188
Searching for meaning	22.50	7.84	−0.476	−0.524
Depression	12.73	7.20	0.680	−0.338

**Table 2:** Pearson correlation coefficients between anxiety, presence of meaning, searching for meaning, and depression (*N* = 277).

	Anxiety	Presence of Meaning	Searching for Meaning	Depression
Anxiety	1			
Presence of meaning	−0.30 ***	1		
Searching for meaning	0.21 ***	0.34 ***	1	
Depression	0.62 ***	−0.31 ***	0.20 ***	1

*** *p* < 0.001.

## Data Availability

The datasets used during this study are available from the corresponding author.
